# Addition of Coffee Waste-Derived Plasticizer Improves Processability and Barrier Properties of Poly(3-hydroxybutyrate-co-3-hydroxyvalerate)-Natural Rubber Bioplastic

**DOI:** 10.3390/polym16152164

**Published:** 2024-07-30

**Authors:** Rinky Ghosh, Xiaoying Zhao, Yael Vodovotz

**Affiliations:** 1 Department of Food Science and Technology, The Ohio State University, 2015 Fyffe Road, Columbus, OH 43210, USA; ghosh.245@osu.edu; 2 School of Light Industry Science and Engineering, Beijing Technology and Business University, No. 33 Fucheng Road, Beijing 100048, China; zhaoxy@btbu.edu.cn

**Keywords:** PHBV, NR, coffee oil epoxide, extrusion, food waste, packaging, bioplastic, scale-up

## Abstract

This study aimed to develop a value-added bio-based polymer product for food packaging. Poly(3-hydroxybutyrate-co-3-hydroxyvalerate) (PHBV) is a promising bioplastic with limitations in processability and brittleness, which our group previously addressed by incorporating high-molecular-weight natural rubber (NR) compatibilized with peroxide and coagent. Yet, processability in an industrial setting proved difficult. Coffee oil epoxide (COE), a waste-derived plasticizer, was incorporated into the PHBV/NR/peroxide/coagent matrix via extrusion, and properties of resulting sheets were evaluated. COE incorporation significantly decreased the oxygen and water permeability of the PHBV/NR sheets. Maximum degradation temperature T_peak_ (°C) increased by ~4.6 °C, and degree of crystallinity decreased by ~15.5% relative to pristine PHBV, indicating good thermal stability. Melting (T_m_) and glass transition temperatures (T_g_) of the PHBV/NR blend remained unchanged with COE incorporation. X-ray diffraction (XRD) revealed ~10.36% decrease in crystal size for the plasticized blend. Energy-dispersive X-ray analysis (EDAX) and scanning electron microscopy (SEM) confirmed good dispersion with no phase separation. The water uptake capacity of the plasticized blend was reduced by 61.02%, while surface contact angle measurements showed improved water resistance. The plasticized PHBV sheet shows promise for environmentally friendly packaging films due to its high thermal stability, effective barrier properties, and industrial scalability.

## 1. Introduction

Plastics derived from petrochemical sources raise environmental concerns due to their consumption of renewable petroleum resources, limited biodegradability, and persistence in the ecosystem [1]. Currently, 46% of plastic wastes are attributed to packaging material [2]. Nearly 90% of the most prevalent types of plastics utilized in the packaging sector (such as polyethylene, polypropylene, polyethylene terephthalate) are derived from petroleum, non-renewable sources, and can last for decades or even centuries [3]. Biodegradable polymers offer a promising alternative to traditional plastics for food packaging. However, their inherent limitations in barrier properties, mechanical strength, and water resistance often hinder their performance. Nanotechnology offers exciting possibilities to enhance these properties. Studies have demonstrated the effectiveness of incorporating nanoparticles into bio-based films. Rhim et al. [4] reported a significant improvement (50% increase) in the oxygen barrier properties of PLA films by introducing clay kaolinite composites. Similarly, other researchers observed enhanced tensile strength and modulus of elasticity in films containing zeolite type 4 particles [5]. Another study reported by Dash et al. [6] on agar-based films incorporated with zinc oxide (ZnO) nanoparticles demonstrated enhanced packaging properties. These composite films effectively preserved green grapes for up to 21 days due to their significant antibacterial activity, increased water vapor permeability, and improved elongation at break. These findings highlight the potential of nanotechnology in enhancing the performance of biodegradable food packaging materials. However, further research is necessary to address the uncertainties concerning the long-term environmental and health implications of nanoparticle release during degradation. Assessing the bioaccumulation and toxicity of these nanomaterials in the environment and food chain is critical [7].

Poly(3-hydroxybutyrate-co-3-hydroxyvalerate) (PHBV) is a microbially derived copolyester with varied hydroxyvalerate and hydroxybutyrate contents and is one of the few biopolymers with demonstrated biodegradability in both soil and marine environments [8,9,10]. PHBV has a high degree of crystallinity and a slow crystallization rate, which constrains its applications in flexible packaging [11,12]. One of the primary challenges is the potential for premature degradation of the packaging material, which can compromise product integrity and shelf life. Factors such as temperature, humidity, and exposure to microorganisms can accelerate the degradation process, leading to package failure and contamination of the food product [13]. Furthermore, the incorporation of additives to enhance the properties of biodegradable plastics often introduces additional complexities. Some additives may not be chemically bound to the polymer matrix, leading to migration or leaching, which could potentially affect food safety and product quality [14]. PHBV processability is greatly influenced by its thermal and mechanical properties [15,16,17,18] and, thus, is prone to thermal decomposition (narrow processing window) when extruded due to the high temperature, shear rate, and residence time. Several studies have been conducted to enhance the thermal and mechanical characteristics of PHBV by combining it with flexible materials such as poly (butylene adipate-co-terephthalate) (PBAT), poly (butylene succinate) (PBS), and natural rubber (NR) [19,20,21]. Zhang et al. examined the thermal decomposition of pristine PHBV during extrusion processing and found that employing a reverse temperature profile was imperative to prevent thermal degradation [22]. Additionally, NR not only reinforced PHBV but also facilitated its melt processing more effectively compared to commercially available alternatives [23,24]. However, as observed by Tomano et al. [25], the addition of NR as a toughening agent for PHBV often results in a significant reduction in tensile strength, with a potential decrease of up to 80%. This can be primarily ascribed to the relatively low stiffness of NR and the inherent immiscibility between PHBV and NR phases.

Miscibility in polymer blends is crucial for homogeneous mixing and improved thermomechanical properties. Selective compatibilizers, including peroxides and coagents, facilitated PHBV/NR dynamic extrusion [24]. This strategy prevented phase separation and alleviated mechanical issues in immiscible blends, leading to enhanced interfacial adhesion and improved matrix morphology [25]. George et al. [26] used dicumyl peroxide (DCP) as an initiator for dynamic crosslinking in acrylonitrile butadiene rubber (NBR), resulting in a blend of high-density polyethylene (HDPE) and NBR with a consistent and textured morphology. This approach further fostered improved compatibility between the HDPE and NBR components, consequently resulting in enhanced mechanical characteristics. Similarly, polypropylene (PP) and ethylene–propylene rubber (EPR) combined in an 80/20 ratio with a coagent via peroxide-initiated reactive blending resulted in a reduction in PP chain scission [27]. Pilot-scale production of PHBV/NR blend sheets revealed limitations due to phase separation and inherent polymer incompatibility. Efforts to improve miscibility, including compatibilizers and peroxides explored by our group and others, have shown some success, but their impact on final blend properties, especially when scaled up in an industry setting, remains limited [23]. This study emphasizes the need for a deeper understanding of the factors affecting miscibility in PHBV/NR blends. By elucidating the relationship between miscibility and barrier properties, we can develop strategies to optimize PHBV/NR blends for industrial success.

Plasticizers play a significant role in enhancing the flexibility of plastics such as epoxy resins and polyvinyl chloride (PVC) by mitigating polymer interaction [28]. Consequently, plasticizers provide a softening effect by influencing the crystallinity of both synthetic and bio-based polymeric materials [29]. Plasticizers also aid in improving toughness and polymer processability during extrusion [30]. In flexible film manufacturing, PHBV blends are often plasticized to further reduce the glass transition temperature (T_g_), widen their melt processing capability, and impart flexibility to the resulting bioplastics [31]. Nosal et al. (2020) explored fatty acid esters as plasticizers for PHBV, demonstrating a significant increase in impact strength (up to 4.1 kJ/m^2^). Their findings suggested that these plasticizers significantly improved mechanical strength and reduced long-term brittleness, making PHBV a more viable biopolymer for packaging applications [32].

The leaching of certain plasticizers derived from petrochemicals, such as phthalates, poses significant safety risks to health and the environment [33]. Due to the lack of chemical bonding between plasticizers and the polymeric matrix, there is a degree of plasticizer loss due to leaching or migration during usage or aging, resulting in a spectrum of issues, i.e., from discoloration and stress cracking to fogging [34]. For instance, certain compounds such as butyl benzyl phthalate (BBP), di-n-butyl phthalate (DBP), and di-(2-ethylhexyl) phthalate (DEHP), traditionally employed as softeners and plasticizers in toys, packaging products, and the polyvinyl chloride (PVC) industry, have been recognized as substances that can disrupt the endocrine system [35]. To overcome these drawbacks, it is necessary to incorporate alternative plasticizers derived from renewable and bio-based sources. Vegetable oils, such as rapeseed and sunflower oils, emerge as promising substitutes for synthetic esters and conventional lubricating oils [36,37]. These alternative plasticizers offer advantages such as a high viscosity index, lower toxicity, favorable lubricity, reduced volatility, cost-effectiveness, renewability, and biodegradability [38]. Vegetable oils contain C-C unsaturated bonds, reducing both oxidative and thermal stability [39], restricting their use as a lubricant to a very narrow temperature range. The direct utilization of epoxidized vegetable oils in the field of packaging materials exhibits remarkable prospects due to its excellent efficiency of plasticization [40]. Epoxidized vegetable oils demonstrate a pronounced toughening effect through grafting or blending modifications and have diverse industrial applications [41,42,43]. The use of epoxidized soybean oil (ESO) as a plasticizer in polyvinyl alcohol (PVA) emulsions, chlorinated rubber, and PVC compounds was reported by Choi et al. [44]. As an alternative, the use of an oil derived from a waste product, such as spent coffee grounds, may prove more appropriate [45].

Approximately 90% of coffee beans are estimated to end up in landfills as discarded spent coffee grounds (SCGs) [46]. The oil content in coffee beans varies between 11% and 20% by weight, depending on the coffee type [47], and, therefore, SCGs serve as a potential plasticizer and internal lubricating composites, thereby improving the mixing process [48]. Moreover, they constitute coffee oil, primarily composed of triacylglycerols [49] similar to those found in vegetable oils. Most studies focused on materials prepared through the solvent casting method on a lab scale, providing limited or no information on the plasticization efficiency of PHBV blends when employing reactive extrusion for material preparation. This method is the most conventional approach for shaping plastics on an industrial scale [50,51,52,53,54].

**Figure 1:**
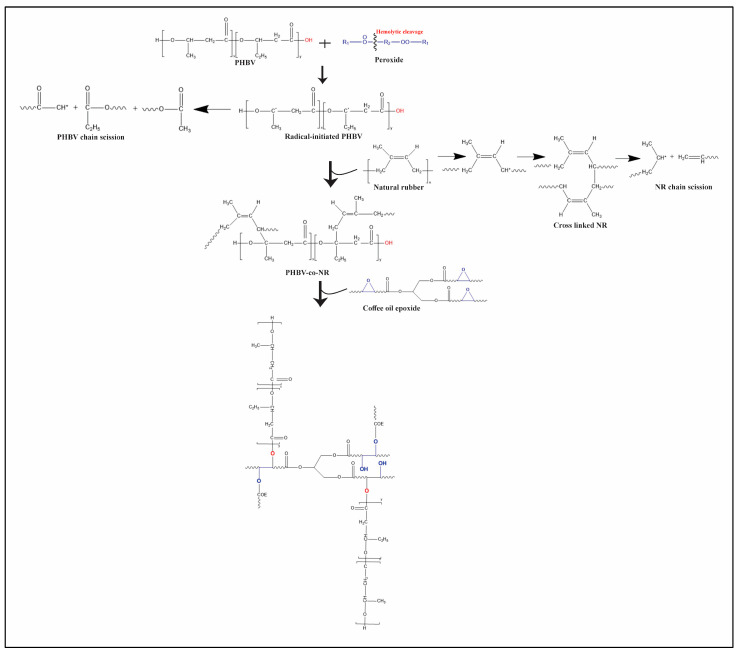
Schematic simplified pathways of relevant reactions involved in Poly(3-hydroxybutyrate-co-3-hydroxyvalerate) (PHBV), Natural Rubber (NR), and coffee oil epoxide via melt processing technique.

The objective of this study was to investigate the effect of employing coffee oil epoxide as a plasticizer for PHBV/NR/peroxide/coagent blends in high melt processing using a twin-screw extruder (Figure 1). The previous research findings revealed that this blend exhibited enhanced mechanical, thermal, and rheological properties compared to pure PHBV, making it well-suited for thermoformed packaging applications. Despite promising results on a pilot scale, PHBV/NR/peroxide/coagent blends failed on an industrial scale due to stiffness during sheet extrusion, potentially due to high melt strength and increased viscosity of the polymer melt, making it difficult to create a continuous web. However, we hypothesize that the addition of a waste-derived plasticizer will enhance the machining of this bio-based blend, creating a sheet on an industrial scale that has improved barrier properties and thermal stability as compared to samples without plasticizer. Therefore, the present work is focused on the measurement of permeability, chemical and thermal properties of plasticized and non-plasticized blends to assess their suitability for food packaging applications. The future work will involve mechanical properties and the evaluation of the leaching behavior of the plasticized blends in various food simulants.

## 2. Experimental Section

### 2.1. Materials

**Figure 2:**
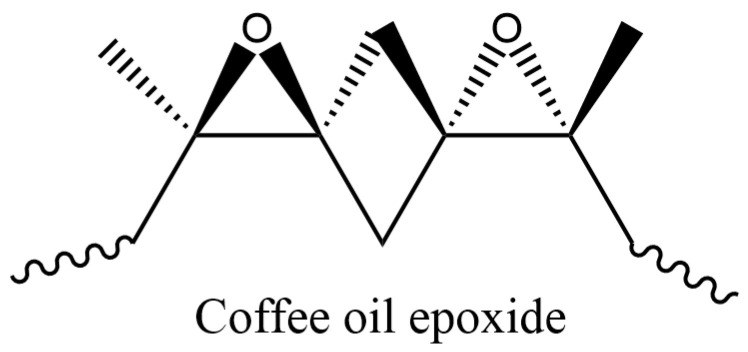
Structure of coffee oil epoxide.

The materials selected for the present studies were PHBV granules (hydroxy valerate (HV) content: 2 mol%) procured from Tianan Biological Material Co. (Ningbo, China). The co-agent trimethylolpropane triacrylate (TMPTA) and 2, 5-Bis (tert-butylperoxy)-2, 5-dimethylhexane, 92% (Peroxide Luperox 101XL45, molecular weight: 290.44), were procured from Sigma-Aldrich (St. Louis, MO, USA) and Thermo Fisher SCIENTIFIC, NJ, USA. NR was purchased from Midwest Elastomers INC. The COE (Figure 2) utilized in this study was obtained as discussed in Williamson et al. [55].

### 2.2. Preparation of Plasticized Blends

To mitigate the risk of hydrolytic decomposition during subsequent melt processing, the PHBV granules were vacuum-dried at 60 °C for 24 h. The moisture content of the blends was determined to be 0.1%. The blends were produced by combining PHBV and NR with an optimal composition including 0.45 wt% peroxide, 0.63 wt% coagent, 85 wt% PHBV, 15 wt% NR, and 0.3 wt% COE [24]. The PHBV blends were processed in a Leistritz ZSE-27 twin-screw extruder (Allendale, NJ, USA), with a screw diameter of 27 mm and an L/D ratio of 40:1. The extruder operated with a reverse compounding temperature of 180 °C to 160 °C. The screw profile (with a screw speed of 64 rpm and a flow rate of ~10 kg/h) was fine-tuned to reduce thermal decomposition during the extrusion process (Table 1). Finally, the extrudate emerged from the die (melt pushed out of the end of the extruder) and was promptly cut into pellets (feed roller speed ~2–3 rpm) using a pelletizer (Scheer Bay Co., Bay, MI, USA). Furthermore, to produce sheets of PHBV/NR and PHBV/NR/COE blends, the PHBV/NR pellets were reintroduced into an extruder equipped with a 3-layer multifold die (MuCell Extrusion LLC, Woburn, MA, USA). The initial temperature profile followed standard settings, with temperature ranging from 190 °C to 202 °C, which were then adjusted by lowering temperatures by 10 °C across the extruder and die to increase melt viscosity. The sheeting die gap was set to approximately 0.51 to 0.89 mm. The sheet obtained from the die was fed through a roll stand equipped with water cooling, maintaining room temperature rolls to prevent sticking and ensure consistent sheet gauge and surface quality. The thickness of sheet is significantly influenced by various processing parameters, including temperature, feed roller velocity, and the speed at which the extruder’s screw rotates. To ensure experimental integrity, all variables were kept constant throughout the conducted experiments. In this study, the PHBV/NR blend treated with coagent and peroxide is referred to as PHBV/NR. The blend plasticized with COE is denoted as PHBV/NR/COE.

## 3. Characterization of Plasticized Sheets

### 3.1. Thermal Characterization

#### 3.1.1. Differential Scanning Calorimetry-DSC

The thermal behavior of PHBV/NR (unplasticized) and PHBV/NR/COE (plasticized) sheets was investigated utilizing differential scanning calorimetry (DSC2500, TA Instruments, New Castle, DE, USA) to analyze the relative crystallization and melting behavior of the samples. Prior to testing, the samples were vacuum-dried at 60 °C for 24 h. Sample sizes ranging from 5 to 10 mg were sealed in a T_zero_ pan and were subjected to DSC testing. Thermograms were obtained using a method that has been previously demonstrated by Modi et al. [56], i.e., heating the sample from −20 to 200 °C at a rate of 10 °C/min, enabling the determination of melting transitions. Upon reaching 200 °C, the samples were isothermally held for 5 min to eliminate any previous thermal effects. The sample was then cooled to −85 °C at a rate of 10 °C/min, held for 5 min before being reheated to 200 °C at the same rate. The degree of crystallinity for the blends was assessed by comparing the enthalpy value obtained from the melting endotherm of the blends with the enthalpy value of a pure PHBV, which has 100% crystallinity (146 J/g), as reported in the literature [57,58]. To ensure precision of the test, triplicates of each blended sheet were considered.

#### 3.1.2. Thermal Degradation Analysis-TGA

The thermogravimetric analysis (TA Instruments, New Castle, DE, USA) was used to evaluate the thermal stability of the PHBV/NR and PHBV/NR/COE blend. The specimens were gradually heated in a nitrogen atmosphere from ambient temperature to 500 °C at a rate of 20 °C/min. TRIOS Software v4.1.1 was used to analyze the weight loss and derivative thermograms (DTG) and to calculate the degradation onset temperature (T_onset_) and peak temperature (T_peak_). At least five replicates for each sample were examined, and the values are reported as average ± standard deviation.

### 3.2. X-ray Diffraction (XRD)

X-ray diffraction (XRD) was employed to characterize the crystalline structure of the PHBV blended sheets. A Rigaku Miniflex 600 diffractometer (Woodlands, TX, USA) equipped with CuKα radiation (λ = 1.5406 Å) was used. The operating parameters of the diffractometer were set at 40 kV and 15 mA. The diffraction patterns were collected over a 2θ range of 5° to 60° with a scanning rate of 2° min^−1^. To ensure consistency and minimize experimental error, all XRD measurements were performed at room temperature under identical operational conditions. The analysis for each sample was repeated three times to improve the data’s validity.

### 3.3. Permeability Testing

#### 3.3.1. Oxygen Transmission Rate (OTR)

The OTR of the PHBV blended sheets was determined following the ASTM D 3985 standard using an AMETEK MOCON OX-TRAN model 2/12 OTR instrument (Minneapolis, MN, USA) [59]. The tests were conducted on extruded sheets with a thickness of 16 mils at 23 °C and a relative humidity of 0%. The testing area of the samples was determined to be 19.62 cm^2^, and a permeant gas consisting of 100% oxygen concentration was utilized for the measurements [60]. The experiments were conducted in triplicate, and the reported values include averages and standard deviations.

#### 3.3.2. Water Vapor Transmission Rate (WVTR)

The WVTR of the PHBV/NR and PHBV/NR/COE blended sheets was investigated following the ASTM E96/E96M-16 standard method [61], utilizing a dynamic vapor sorption instrument manufactured by Surface Measurement Systems Ltd. (Allentown, PA, USA). The instrument was set to a standard temperature of 40 °C for a duration of 24 h. To assess moisture transport, a Payne cell with an opening area of 15.54 mm^2^ was employed [62]. The sheets of each composition were cut in a circular form with the help of scissors, maintaining an average diameter of 7 mm and thickness of ∼0.1 mm, mounted on top of the Payne cell. The film-sealed Payne cell was filled with a drying agent, ensuring that the humidity levels inside and outside of the cell were maintained at 0% (desiccant) and 90% relative humidity (RH), respectively. The data correspond to the average of three tests.

Water vapor transmission rate (g/24 h/m^2^) was calculated using the given ASTM formula (Equation (1)):

(1) $$WVTR=\frac{\;\mathrm{Slope}\;\mathrm{of}\;\mathrm{the}\;\mathrm{curve}\;(G)}{\mathrm{Time}\;(t)\times \mathrm{planar}\;\mathrm{area}\;\mathrm{of}\;\mathrm{film}}$$

### 3.4. Water Uptake Capacity

The determination of water absorption capacity of the PHBV/NR and PHBV/NR/COE blended sheets was conducted as per the ASTM D570 standard [63]. The blended samples were cut into equal dimensions of 15 mm × 15 mm and subsequently evaluated in distilled water. At the start of the experiment, the initial weight (Wd) of the samples was measured using a digital microbalance. Subsequently, the samples were promptly submerged in 10 mL of water and subjected to a temperature of 37 °C for a duration of 4 days until they achieved equilibrium. During the prescribed soaking period, wet samples were periodically extracted from the water every 24 h. These samples were then carefully wiped using tissue paper to eliminate any surplus water, weighed (W_w_), and subsequently placed back into the water. The water absorption capacity can be quantified by calculating the ratio of the difference between the final weight and the initial weight (numerator) divided by the initial weight (denominator). To determine the mechanism underlying the water uptake capacity of the blended sheets, various kinetic models were employed. The models either explain the mechanism in terms of chemical adsorption, namely, pseudo-first-order and -second-order models or in terms of physical adsorption, namely, intraparticle diffusion model [64].

The initial phase of adsorption, given by (Equation (2)), is described by the pseudo-first-order model:

(2) $$\log(q_{e}-q_{t})=logq_{e}-\frac{{\;k}_{1}t}{2.303}$$

The parameters *q_t_* and *q_e_* denote amount of water adsorbed at time *t* and at equilibrium, respectively. By plotting *log*(*q_e_* − *q_t_*) against time (*t*), the slope of the resulting graph provides information about the rate constant (*k*_1_), while the intercept corresponds to *log*(*q_e_*).

The mathematical equation that represents the pseudo-second-order model is given by (Equation (3)).

(3) $$\frac{t}{q_{t}}=\frac{1}{{k_{2}q_{e}}^{2}}+\frac{t}{q_{e}}$$

where *k*_2_ is the rate constant. The parameters in (Equation (3)) can be determined through graphical representation of time (*t*) against the reciprocal of adsorption quantity (*t*/q*_t_*). In this graphical representation, the slope and intercept are represented by *q_e_* and *k*_2_, respectively.

The intraparticle adsorption–diffusion model can be represented by (Equation (4)):

(4) $$q_{t}=k_{in}\sqrt{t}+C$$

Here, *q_t_* represents the quantity of water that has been adsorbed at a given time “*t*”. The kinetic constant *k_in_* values are associated with the intraparticle diffusion parameter. *C* is indicative of the boundary layer thickness. A plot of *q_t_* versus $\sqrt{t}$, where *k_in_* represent the slope and *C* denotes the intercept, can be used to determine the values of the parameter’s *k_in_* and *C*. The present model provides a description of the kinetics of water adsorption in the PHBV/NR and PHBV/NR/COE blended sheet. The tests were conducted in triplicate.

### 3.5. Water Contact Angle

In this research study, the KRÜSS DSA 100 goniometer (Hamburg, Germany) was employed to conduct contact angle and surface tension measurements [65]. The sessile drop method was utilized to determine the sliding angles, while the pendant drop method was employed to calculate the interfacial tensions. The sessile drop method involved measuring the angle at which a droplet begins to slide on the surface, while the pendant drop method analyzed the shape of a suspended droplet to estimate interfacial tensions or forces. To ensure accurate results, a slow flow rate of 5 μL/min was maintained during the measurements to minimize the impact of dynamic forces on the droplet’s shape. A Linkam PE120 Peltier hot stage was employed to maintain precise temperature control throughout the experiments. To determine the contact angles and sliding angles, 3 μL water droplets were deposited onto the surface using the liquid dispensing function of the goniometer. For sliding angle measurements, the goniometer was gradually tilted at a rate of 1°/min until the droplet initiated sliding. High-resolution images of these droplets were captured using a camera and analyzed using integrated software to calculate the surface and interfacial tensions. Contact angle measurements were conducted on three replicates for each sample.

### 3.6. Nuclear Magnetic Resonance (NMR)

High-resolution one-dimensional nuclear magnetic resonance (^1^H NMR) spectra were acquired in a 5 mm NMR glass tube at ambient temperature using a Bruker Fourier 80 MHz NMR spectrometer (Billerica, MA, USA). The spectral data were processed with the Topspin 4.2.0 software package from Brüker Biospin. For ^1^H NMR analysis, samples were prepared in deuterated chloroform (CDCl_3_) at a concentration of 10 mg/mL. Spectra were recorded with a 90° pulse angle, a pulse duration of 10.37 μs, and a 5 s delay time. The residual proton peak of CDCl_3_ at 7.26 ppm was used as a chemical shift reference.

### 3.7. Scanning Electron Microscopy (SEM) and EDAX

Topographic and compositional imaging of the PHBV, PHBV/NR, and PHBV/NR/COE samples was conducted using scanning electron microscopy SEM (Quattro, Thermo Fisher Scientific, USA). The SEM offers high magnification with a resolution of 3.0 nm at 1 kV or 0.8 nm at 30 kV when operating in STEM mode. The measurements were performed under low vacuum using ETD (Everhart-Thornley Detector) and ABS (Segmented annular backscatter) detectors. The evaluation of the mean diameter of particles in SEM images was conducted utilizing ImageJ software.

Energy-dispersive X-ray analysis (EDAX) measurements were conducted with an ETD detector to examine the elemental composition of the samples. The study was conducted at a working distance of 13.4 mm and an electron production voltage of 5 kV. To prevent electrostatic charging during electron irradiation, vacuum-dried samples were carbon-coated using carbon fiber thread with a diameter of 1.3 mm to achieve a coating thickness of 10 nm, utilizing the Leica ACE600 Sputter Coater (Wetzlar, Germany). For comprehensive analysis, three replicates of the samples were employed in low-vacuum mode during SEM analysis.

## 4. Results and Discussion

### 4.1. Effect of Plasticizer on Crystallization and Melting Behaviors

**Figure 3:**
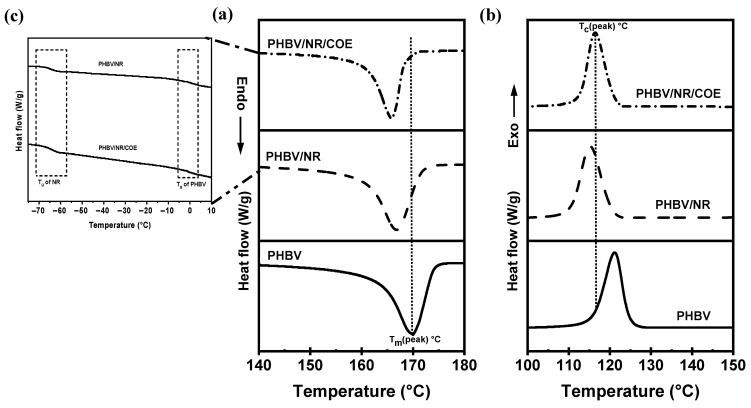
DSC (**a**) heating, (**b**) cooling cycle of PHBV and its NR blend, and (**c**) inset of the curve region depicting T_g_ of NR and PHBV in the blend.

Figure 3 depicts the DSC thermograms of PHBV and the blended sheets at 10 °C/min scan rate. The melting of the samples was followed by three distinct crystallization regimes for all the compositions (Figure 3a,b). During the first heating scan, a distinctive exothermic peak corresponding to cold crystallization was observed for all the samples (Table 2). The crystallization temperature (T_c_) of the PHBV/NR/COE sheets was suppressed by 4° from ~124.5 °C to ~119.8 °C, compared to pristine PHBV. However, the crystallization enthalpy (ΔH_c_) remained comparable for the PHBV/NR/COE and PHBV/NR blends. The decrease in cold crystallization temperature compared to neat PHBV indicates the presence of a crystallite population with reduced lamellar thickness [66]. Furthermore, the crystalline fraction, determined from the melting enthalpies of the blends, demonstrates a decrease with the introduction of NR. The incorporation of NR results in the formation of larger rubber droplets (as evidenced by SEM images) compared to the PHBV/NR/COE blend. This, in turn, leads to an overall reduction in the interfacial contact area between the PHBV matrix and the rubber phase [57]. These findings highlight the intricate relationship between the cold crystallization behavior of the blends, their morphology, rubber content, and interfacial characteristics. Moreover, the incorporation of COE resulted in a synergistic effect that enhances the mobility of the polymeric chain at the phase boundary and was suppressed to promote chain growth during crystallization. As a result, the addition of plasticizer slightly decreased the crystallinity (by 1%) and decreased the glass transition temperature (T_g_) of the PHBV/NR blend components (by ~1 °C), indicating improved miscibility and mobility of the polymer chains in the amorphous phase (Figure 3c). This aligns with the free volume theory, which predicts an increase in free volume within the polymeric matrix upon plasticizer addition [67]. Similar observations of reduced crystallinity and T_g_ with plasticizer addition have been reported by Azuma et al. [68] and others using epoxidized vegetable oil for PHBV [69]. It is hypothesized that subsequent higher loadings of the COE plasticizer might lead to a more significant effect on both T_g_ and crystallinity. Interestingly, the melting temperature (T_m_) of the PHBV/NR and PHBV/NR/COE blends mostly remained unchanged with the incorporation of COE plasticizer. It suggests that the plasticizer’s effect is localized in the amorphous regions, but there is no significant change in lamella thickness.

### 4.2. Effect of Plasticizer on Thermal Stability of PHBV/NR Blended Sheets

**Figure 4:**
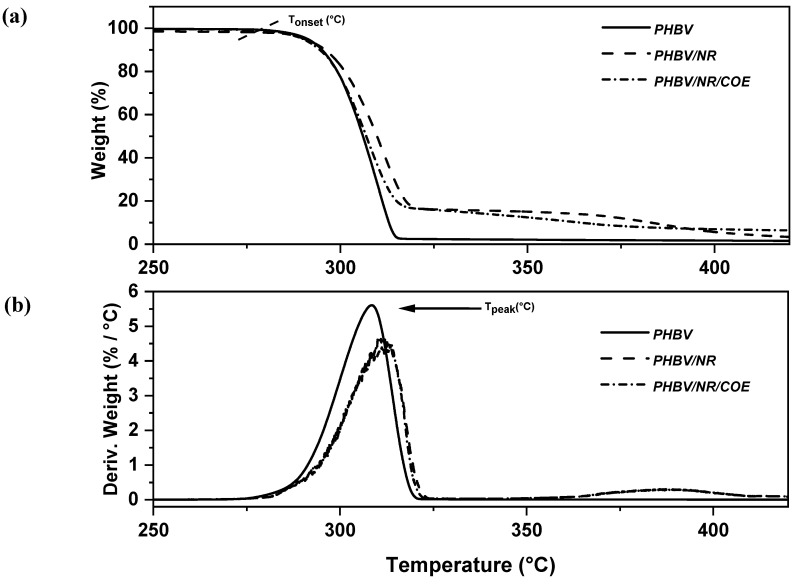
Thermal stability of PHBV and its blend, (**a**) TGA thermograms, and (**b**) derivative weight thermograms.

The thermal degradation profiles of PHBV and its blends (Figure 4) revealed an onset of decomposition (T_onset_) at approximately 290–300 °C, reaching a peak degradation temperature (T_peak_) around 300–310 °C, aligning with previous findings [23,24]. Interestingly, all blends exhibited two distinct weight loss stages in the TGA curves (Figure 4a), suggesting degradation products with varying thermal stabilities. The first derivative curves (Figure 4b) indicated enhanced compatibility between PHBV and NR in the presence of COE. This is evidenced by the less prominent minor degradation peak observed in the PHBV/NR/COE blend within the temperature range of 327 °C to 392 °C compared to PHBV/NR. Similar observations have been reported for PLA/PHBV blends prepared via melt processing [70]. Furthermore, no significant changes in the peak degradation temperature were observed for the PHBV/NR and PHBV/NR/COE blends (Table 3). This indicates that the incorporation of COE does not affect the overall thermal degradation behavior of these blends under the studied conditions.

### 4.3. Effect of Plasticizer on Crystalline Structure of PHBV/NR Blended Sheets

**Figure 5:**
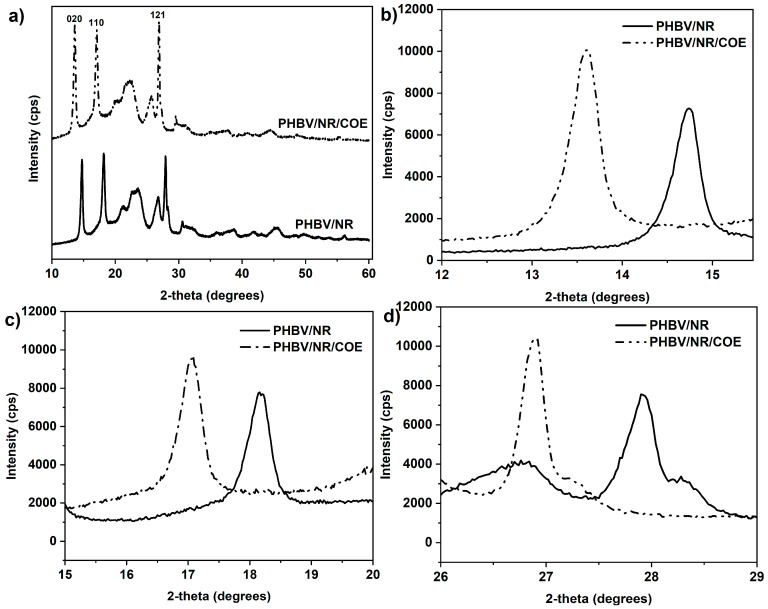
(**a**) XRD Patterns, and (**b**–**d**) Relative Intensities of PHBV/NR and PHBV/NR/COE Blends.

X-ray diffraction measurements were performed to determine the crystalline structure for the PHBV blended samples. The addition of plasticizers could potentially result in a reduction in spherulitic size. This is supported by previous research by Branciforti et al. [71] which suggests a strong influence of plasticizers on the crystal lattice parameters. The XRD revealed several distinct peaks corresponding to specific crystallographic planes in the PHBV sample (Figure 5a). The prominent peaks were observed for the PHBV/NR sheet at approximately 2θ = 14.74°, 18.16°, and 27.9°. The PHBV/NR/COE blended sheet exhibited peaks at 13.6°, 17.08°, and 26.92°. These peaks can be assigned to Miller indices of (020), (110), and (121), respectively, which are indicative of an orthorhombic unit cell [57]. This pattern closely matched previously reported results for PHBV, especially considering the use of Cobalt Kα radiation in this experiment [57].

The addition of plasticizer exhibited limited influence on both the peak intensity and interplanar spacing (d-spacing) of the PHBV/NR blends Figure 5b–d. This suggests changes in the helical torsion angles of the PHBV chains, likely due to weak additive interactions [72]. Moreover, no new peaks were observed within the 2θ range of 5° to 60° for the blended sheets. This finding indicates that COE addition does not induce the formation of new crystalline phases. However, the peaks corresponding to (020), (110), and (121) Miller indices exhibited a shift towards higher angles in the PHBV/NR blended sheets compared to the plasticized sheet. The observed increase in d-spacing (as shown in Table 4) and narrowing of characteristic reflections might be attributed to the formation of more perfect crystals with improved long-range order, potentially leading to a corresponding increase in spherulitic size upon plasticizer addition. The plasticizer might also improve the mobility of polymer chains, allowing them to rearrange more efficiently and pack into smaller crystalline structures [71]. Despite the potentially increased free volume attributed to COE’s longer chain structure, the current findings indicate that the employed concentration of COE might not be sufficient to achieve effective plasticization. In this initial study, we focused on a specific concentration to thoroughly evaluate its effects on the PHBV/NR blend. We are currently investigating the effects of various concentrations of COE to provide a more comprehensive understanding.

The degree of crystallinity (*X_c_*) within the blended sheets was determined using Equation (5) (details provided in the Table 4) [73]. Here, A_c_ and A_A_ represent the areas under the crystalline and amorphous peaks, respectively. This calculation involved the ratio of the integrated areas for the five crystalline peaks to the total area of all peaks in the XRD pattern. This approach provides a more accurate representation of the intrinsic crystallinity within the blends, allowing for a better comparison across compositions with varying ratios of components. Interestingly, the crystallinity of the PHBV/NR/COE blend (68.85%) was found to be higher compared to the PHBV/NR blend (63.52%). Although the addition of plasticizer led to a slight increase in the degree of crystallinity, as indicated by the minor shifts in Bragg angles and the observed increase in interplanar distances, this variation seems to be within experimental error. Therefore, the plasticizer appears to have minimal influence on the overall crystallinity of the blends.

(5) $$X_{c}\%=\left[\frac{A_{c}}{A_{C}+A_{A}}\right]\times 100$$

To gain further insight into the influence of plasticizer on the crystallinity of the PHBV/NR blend, the Scherrer equation (Equation (6)) was employed to analyze the Full Width at Half Maximum (FWHM) of the diffraction peaks in the XRD pattern [71]. Therefore, an observed increase in FWHM would signify a decrease in the average crystallite size within the blend upon COE plasticizer incorporation. This finding aligns with the observed minimal change in overall crystallinity (discussed earlier) and suggests that the plasticizer might primarily affect the perfection and size of individual crystallites within the PHBV/NR matrix, rather than significantly altering the overall crystalline fraction.

(6) D=kλ/βcosθ

The X-ray wavelength (λ = 1.5406 Å), the scattering angle (θ), the full width at half-maximum (FWHM) of the peak (β), and the Scherrer constant (K = 0.9) are some of the characteristics that can be used to calculate the crystal size (*D*).

The strongest scattering peak (020) in the PHBV/NR blend exhibited a D-value of 22.67 nm compared to 20.32 nm for the plasticized blend (PHBV/NR/COE). The potential role of COE as a nucleating agent could explain the formation of more numerous, smaller crystallites within the blend. This would lead to a decrease in D size and an increase in the total crystalline fraction. This observed increase in crystallinity from XRD data, however, appeared to contradict the slight decrease in crystallinity measured by DSC analysis. This discrepancy highlights the limitations of each technique. XRD primarily probes the crystalline fraction within the material, while DSC measures the overall enthalpic changes associated with melting processes. Further detailed discussion of the distribution of the COE phase and its influence on the crystallinity of PHBV/NR blend will be elucidated in the morphological section of this study.

### 4.4. Effect of Plasticizer on Water Permeability of the PHBV/NR Blend

**Figure 6:**
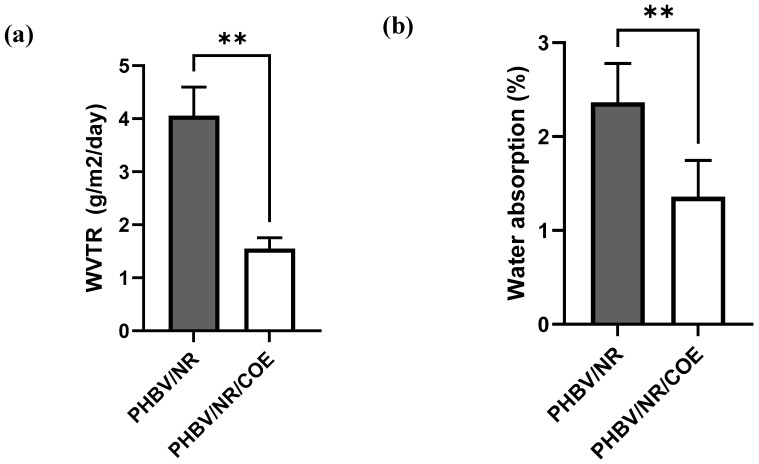
Permeability measurements: (**a**) WVTR, (**b**) water uptake capacity of the blended system. The notation ** (*p* < 0.05) denotes statistical significance and indicates a significant difference from the control group.

WVTR values were evaluated for all samples, and results are depicted in Figure 6a. The values obtained for PHBV blends in this study are consistent with those reported in the previous literature [24]. The addition of 15 wt% rubber contents in PHBV/NR, facilitated by the presence of compatibilizers, resulted in a significant decrease in WVTR values [24]. A crucial factor influencing the moisture barrier characteristic of polymeric blend is found to be the degree of crystallinity. As the χ_c_ value (crystallinity percentage) increases, the polymer exhibits a greater potential for effective barrier performance. Diez-Pascual et al. found that the final barrier properties of the material are influenced by a variety of factors, such as crystallinity, dispersion state, and filler concentration [74]. In our previous studies, dedicated to the preparation and characterization of PHBV/NR blend using the extrusion method, we observed similar WVTR values [24]. It was concluded that this improvement in barrier properties is attributed to the effective dispersion of rubber within the PHBV phase, leading to increased tortuosity. Additionally, higher barrier properties were associated with the intrinsic low water permeability characteristic of NR. Further investigations suggested that incorporation of NR to the PHBV matrix resulted in strong interfacial interactions which successfully led to denser film structure and thereby improved the water barrier property. Considering the well-established influence of plasticizers in increasing the free volume within polymeric structures and subsequently promoting diffusion rates, it is prudent to anticipate that the addition of a suitable plasticizer would correspondingly lead to enhanced permeabilities within the blend system [75,76]. However, the incorporation of more hydrophobic COE in the PHBV/NR polymer matrix was anticipated to enhance its barrier characteristics by establishing a more tortuous and longer pathway for water and oxygen (O_2_) molecules. A PHBV/NR/COE blend with a WVTR of (1.55 ± 0.2 g/m^2^.24 h) had a better water vapor barrier compared to PHBV/NR blends (WVTR = 4.05 ± 0.54 g/m^2^.24 h). Our findings (see Figure 6a) demonstrated that this type of plasticizer used has a substantial impact on the WVTR of the material. The differences in standard deviation observed could potentially arise from variations in film density and thickness across the samples. The PHBV/NR/COE blend demonstrated a moisture barrier that is comparable to commercially available plastics such as ethylene vinyl acetate (EVA), suggesting its potential suitability for prospective film packaging application [77].

### 4.5. Water Absorption Analysis of PHBV/NR and PHBV/NR/COE Blends

PHBV/NR reached its equilibrium water uptake stage more rapidly (approximately after 50 h) compared to the PHBV/NR/COE blend, which can be ascribed to the more facile diffusion of absorbed water in the PHBV/NR blend without COE content. This behavior is coherent with the higher wettability of the PHBV/NR blend (shown by contact angle measurements) [64]. Additionally, the reduced phase separation and improved miscibility in PHBV/NR/COE blends might decrease interaction with water molecules due to increased interactions within the blend, potentially improving the interfacial adhesion between NR and the PHBV matrix.

An equilibrium water uptake of 3.54% for PHBV/NR and 1.36% for PHBV/NR/COE blend was observed (Figure 6b). The reduction in water absorption could be ascribed to the presence of long hydrophobic alkyl chains in COE plasticizer, which hinders a significant amount of water from entering the PHBV matrix. Additionally, the equilibrium water uptake is ~62% higher for the PHBV/NR blend compared to those blended with COE, creating a hydrophobic barrier that restricted water molecule diffusion within the material. These findings align with the contact angle measurements of the blended sheets. Upon reaching the saturation stage, the absorption rate of the samples decelerated, eventually reaching a state of equilibrium. At this stage, any additional water presence within the void structure does not lead to further expansion, as it exists as unbound (not mechanically entrapped) water.

**Figure 7:**
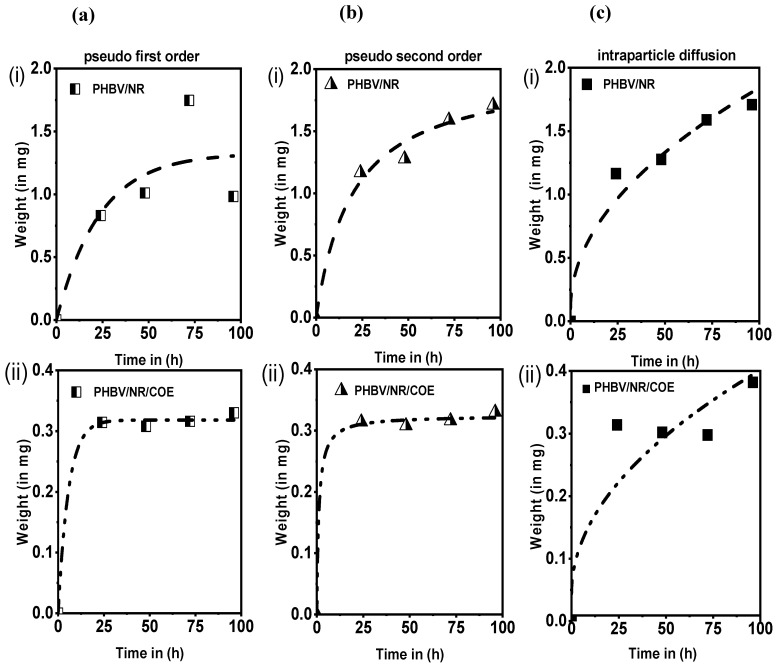
Plots representing the water uptake kinetics: (**a**) pseudo-first-order kinetic model; (**b**) pseudo-second-order kinetic model; (**c**) intraparticle diffusion model for PHBVNR and PHBV/NR/COE sheets.

To elucidate the most appropriate model describing water absorption kinetics, we employed linearized regression analysis of different kinetic models. The experimental data were fitted to established kinetic equations proposed by Ho and McKay for three models: pseudo-first-order (PFO), pseudo-second-order (PSO), and intraparticle diffusion [78,79]. The corresponding Figure 7a–c illustrate these fits. The associated rate constant and equilibrium water uptake derived from these models have been summarized in (Table 5). These results indicate that both the PFO and PSO models effectively describe the water uptake kinetics for both the blends. The consistency between the models’ predictions and experimental data emphasizes the applicability of these models in capturing the dynamic adsorption behavior of water molecules within the blended polymeric matrices.

Analysis of the data revealed that the pseudo-first-order (PFO) model provided the best fit for the PHBV/NR/COE blend, with a correlation coefficient (R^2^) exceeding 0.99. This suggests that the PFO model accurately reflects the physiological process of water uptake, where water molecules penetrate the polymeric sheet through pores, particularly at the observed equilibrium time of 50 h. Interestingly, the equilibrium water absorption q_e_ significantly decreased from 1.38 mg in the PHBV/NR sample to 0.38 mg in the PHBV/NR/COE sample upon COE plasticizer incorporation. This aligns with the observed slower rate constant and suggests that the introduction of hydrophobic COE molecules into the PHBV/NR matrix hinders water uptake. This shift suggests a potential transition towards a chemisorption-like process, where the hydrophobic alkyl chains of COE might interact with the polymer matrix, leading to more stable and less reversible water molecule adsorption. Thus, the presence of COE reduces the film’s affinity for water, making it take longer for the sheets to reach equilibrium with water.

On the contrary, the PSO model effectively describes water uptake in the PHBV/NR blend, as evidenced by the strong correlation coefficients. This is supported by the strong correlation coefficients obtained for both materials, indicating a good fit between the model and the experimental data. Interestingly, the PHBV/NR blend exhibited a particularly high rate constant (k_2_) and equilibrium water uptake (q_e_). These observations suggest a dominant chemisorption phenomenon for this blend, where stronger chemical forces are involved in the interaction between water molecules and the polymer matrix. Our findings support the work of Ho and McKay [79], where a strong correlation coefficient obtained from the linear pseudo-first-order (PSO) model for the PHBV/NR sheet indicates a higher chance of electron exchange between the liquid water and the solid matrix of the sheet [80]. Furthermore, the data suggest that intermolecular diffusion occurs in all the blended sheet samples. However, further investigation is needed to definitively confirm the role of electron exchange in the adsorption process.

### 4.6. Impact of Plasticizer on the Barrier Properties of the PHBV/NR Blends

The permeability of packaging materials to oxygen significantly impacts their utility in food preservation. This is because oxygen migration can facilitate food oxidation, leading to quality degradation in certain products. Surface hydrophobicity plays a key role in the chemical basis of moisture barrier properties. In general, factors including packing density, crystallinity, chemical structure, and affinity affect the morphology of blended polymeric sheets, which in turn affects their permeability and barrier qualities [81]. The OTR of the PHBV/NR and PHBV/NR/COE blended sheets are shown in Table 6. The decrease in OTR value by ~22 cc/m^2^/day upon addition of COE is associated with the hydrophobic nature of the plasticizer used. The OTR of the PHBV/NR sheet was ~2025 cc/m^2^/day. This shows that the bioplasticizer containing long aliphatic groups enhanced the films’ resistance to oxygen transmission. This effect is likely due to the bulky aliphatic groups filling in microscopic voids within the polymer matrix, leading to improved barrier properties [82]. Hydrophobic materials are composed of molecules with a strong resistance to water (and other polar molecules like oxygen). These molecules interact with each other through weak forces like van der Waals interactions and London dispersion forces. This creates a tightly packed and cohesive network that hinders the movement of oxygen molecules through the material. Furthermore, the tightly packed structure of most hydrophobic materials often creates a labyrinthine network of pathways for gas molecules to navigate. This “tortuous” path length significantly increases the diffusion time for oxygen to permeate through the material [83]. It was interesting to observe that the final OTR value of the PHBV/NR/COE sheet is significantly lower compared to conventional food packaging polymers, such as low-density polyethylene (LDPE) and polypropylene (PP), with a thickness of approximately 0.27–0.33 mm for LDPE and 42.8 µm for PP [84,85,86].

### 4.7. Surface Roughness Measurements

**Figure 8:**
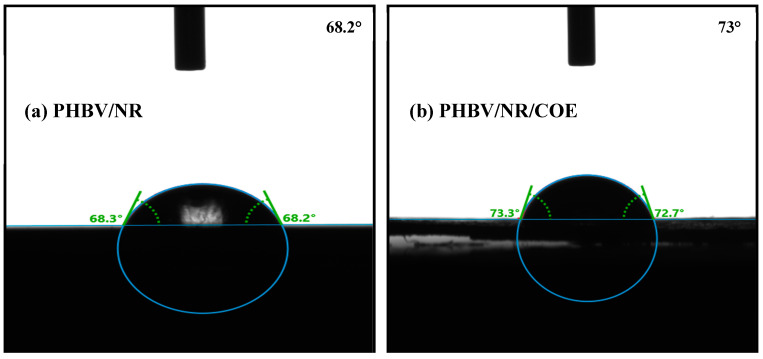
(**a**,**b**) Images depicting water contact angles for the plasticized and non-plasticized blended samples.

The inherent low water resistance capability of bioplastics is inadequate, making their use non-competitive, in comparison to other petro-plastics. Ensuring the preservation of film quality against the detrimental effects of vapor or water exposure can be critical for its practical utilization. As depicted in Figure 8a,b, the inclusion of 0.3% of plasticizer in the PHBV/NR blend had an impact on the wettability and surface energy of the resulting materials. This is reflected in the increased contact angle (CA) of the PHBV/NR/COE film (CA ~71.12°) compared to the PHBV/NR blend (CA ~69.47). Both samples demonstrated a hydrophilic state. However, the incorporation of the COE compound significantly influenced the wettability of the film, causing it to shift towards a more hydrophobic character. Previous studies by Cong et al. [87] have also reported similar effects of surface tension on vegetable oils. The presence of epoxide groups, which possess oxygen atoms capable of interacting with water molecules, is believed to contribute to this hydrophobic behavior. These epoxide groups, being less polar than hydroxyl (OH) groups, exhibit a lower affinity for water, resulting in enhanced hydrophobic characteristics.

This finding aligns with our hypothesis that the incorporation of the coffee oil epoxide plasticizer in the blends would lead to increased contact angles. This effect can be attributed to the improved surface roughness at the microscopic level imparted by the plasticizer. The investigation of plasticized PHBV blends revealed a significant contact angle hysteresis of approximately 20° for the PHBV/NR blend. This hysteresis, reflecting the difference between advancing (θ_a_) and receding (θ_r_) contact angles of a liquid droplet, offers insights into the surface’s interaction with liquids [88]. A larger hysteresis value, as observed here, indicates a less reversible wetting process, suggesting that the liquid droplet encounters greater resistance to spreading and receding on the surface. This hindered movement likely originates from the inherent immiscibility between PHBV and NR. Due to their differing chemical properties, these components tend to separate and form distinct phases within the blend, creating heterogeneity and/or surface roughness. This heterogeneity is further corroborated by SEM topography images, which likely reveal a non-uniform landscape with distinct PHBV and NR domains. This disrupted morphology disrupts the smooth movement of the liquid droplet, leading to a larger hysteresis value.

On the other hand, COE might act as a compatibilizer, promoting better interaction between PHBV and NR, thus leading to a more uniform surface with reduced heterogeneity compared to the PHBV/NR blend alone. This could contribute to a lower hysteresis value despite the increased hydrophobicity (larger contact angle).

### 4.8. Effect of Plasticizers on the Molecular Interactions of the PHBV/NR Blends

**Figure 9:**
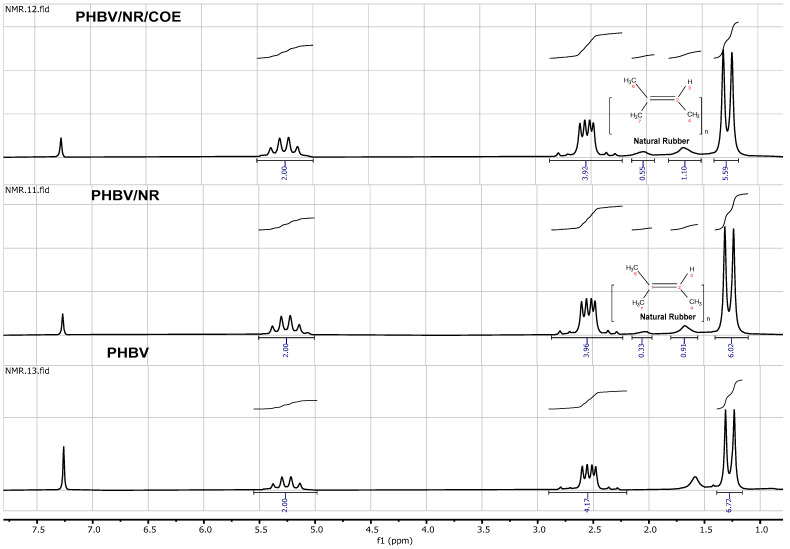
^1^H NMR spectra of PHBV, PHBV/NR, and PHBV/NR/COE, respectively.

**Figure 10:**
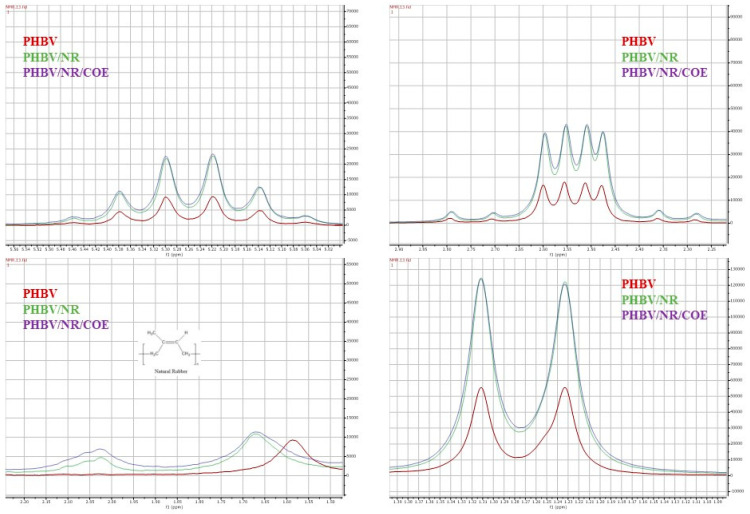
Comparison of Peak Intensities in ¹H NMR Spectra of PHBV, PHBV/NR, and PHBV/NR/COE Blends.

The ^1^H NMR spectra acquired from PHBV/NR and PHBV/NR/COE blends closely resembled that of pristine PHBV (as shown in Figure 9). This observation strongly suggests that the predominant constituents within these samples consist primarily of PHBV. It is imperative to emphasize that there were noticeable differences in the signal intensity (Figure 10). Additionally, the blended system may have subtle differences in composition and intermolecular interactions, as evidenced by these changes in signal strength. ^1^H NMR analysis provided valuable insights into the molecular composition of the blended samples, particularly the methyl group (–CH3), and methylene group (–CH2–) originating from the hydroxyvalerate and hydroxybutyrate monomer units. The chemical shifts observed at 0.89 ppm, 1.32 ppm, 2.38–2.43 ppm, 2.1–2.57 ppm, and 2.59–2.79 ppm correspond to the methyl group of the hydroxybutyrate group, the internal single bond CH2 group of the hydroxyvalerate group, and the single bond CH2 groups, respectively, according to the findings of Gong et al. [89]. The spectra of the blends exhibit characteristic peaks of NR, as depicted in Figure 9. The chemical shift values observed at 2.02 ppm and 1.65 ppm represent the methylene and methyl groups of the NR, respectively [90].

### 4.9. Morphological Properties

**Figure 11:**
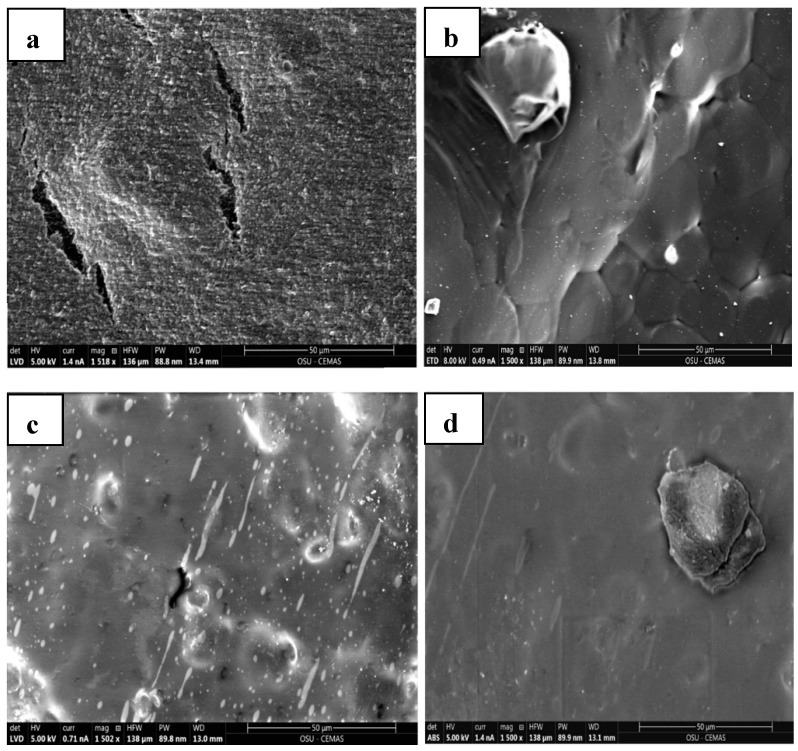
SEM micrographs of (**a**) pristine PHBV, (**b**) PHBV/NR, (**c**) PHBV/NR/COE, and (**d**) PHBV/NR (using ABS detector).

**Figure 12:**
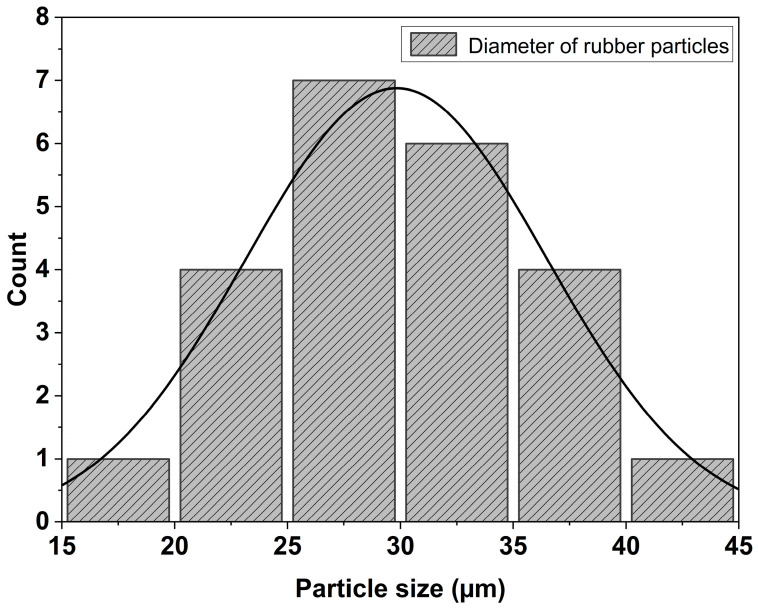
Histogram of the particle size distribution.

Figure 11a depicts the inherent brittleness of the PHBV sheet as observed in the SEM micrographs that had a smooth homogeneous surface with no structural irregularities attributed to the highly oriented structure of the polymeric matrix. But with a gradual addition of the NR, sheets in Figure 11b,c also displayed a smooth surface with ductile characteristics. The observed large rubber droplet size of approximately 29.79 µm in the PHBV/NR blend indicates the presence of an immiscible binary polymeric blend (Figure 12). Conversely, the addition of 0.3% loadings of COE to PHBV/NR blends demonstrated the absence of microdroplets of NR (Figure 11c). Both COE and NR exhibited homogeneous blending with the PHBV matrix, suggesting improved miscibility in comparison to the PHBV/NR blend. This is due to the size fracture supporting good dispersion and an extensive interactive network within the blended system. This phenomenon can be attributed to the process of coalescence of the COE and NR phases within the blended system [72]. The increased roughness/shear surfaces of the sheets suggest a robust interfacial adhesion between the polymeric chains and the NR. Irregularities in surface topography were evidenced by the presence of microcracks in the blended sheet. Therefore, these images illustrate that NR and COE in the plasticized blend are uniformly and finely dispersed in the PHBV phase, forming a network within the bulk matrix of the polymeric sheets; mechanical tests such as tensile testing would be necessary to quantitatively confirm the improved ductility of the blend.

To confirm the identity of the observed clusters in the SEM images, energy-dispersive X-ray analysis (EDAX) was used. The EDAX analysis of the PHBV sample revealed a significantly higher carbon content (86.26%) compared to the PHBV/NR blends (54.61%) in the matrix. This observation suggests that the PHBV matrix is rich in carbon (C). In the context of the PHBV/NR blends, the phenomenon of phase separation results in the segregation of carbon-rich components, potentially associated with natural rubber (NR) or other additives, into discrete regions. This phase separation contributes to variations in carbon content throughout the blended matrix. On the other hand, the introduction of COE plasticizer is observed to enhance uniform blending, evident in an increase in carbon content (75.71%). This implies that the incorporation of COE contributes to a more homogeneous distribution of carbon-rich elements, emphasizing its potential role in achieving uniformity within the blended system.

Furthermore, it is important to mention that the detection of an aluminum (Al) peak in the EDAX spectrum can be attributed to the aluminum coating applied to the sample holder. Moreover, all spectra acquired from regions lacking obvious aggregate clusters consistently displayed a low level of calcium (Ca), magnesium (Mg), and other elements. These elements are likely derived from the calcium-containing peroxide utilized for crosslinking the natural rubber. This study further revealed significant elemental compositional differences between the PHBV/NR and PHBV/NR/COE blends, as shown in Table 7. Higher concentrations of oxygen (O) were observed, suggesting that COE was successfully incorporated into the blend. The increased O content, in comparison to the PHBV/NR blend, can be ascribed to the presence of oxirane rings that arise from the epoxidation of coffee oil, forming a three-membered cyclic ether (Figure 2). This could potentially enhance barrier properties due to the introduction of additional crosslinking or branching within the polymer matrix, improving resistance against gases and other permeants. However, the exact impact on barrier properties would depend on various factors, including the concentration of oxirane rings, their distribution within the blend, and the overall microstructure of the blend.

Further analyses and correlations with material properties will be crucial to elucidate the implications of these compositional differences in the context of the blend’s overall performance and suitability for specific applications.

## 5. Conclusions

This study successfully developed a novel PHBV/NR blend sheet incorporating a coffee waste-derived plasticizer via melt processing. The 0.3 wt% COE significantly improved the blend’s extrusion behavior during scale-up, highlighting its potential as a processing aid derived from a waste resource. Despite minimal influence on overall crystallinity and thermal stability, COE effectively reduced water vapor and oxygen transmission rates, indicating enhanced barrier properties, making these blends promising candidates for packaging applications**.** Future investigations utilizing higher COE concentrations are crucial to definitively assess its role as a plasticizer and its influence on the crystallinity of these blends. Interestingly, XRD analysis revealed a slight increase in d-spacing and crystallinity percentage. However, the plasticizer seems to primarily affect the perfection and size of individual crystallites, evidenced by a decrease in crystallite size (22.67 nm to 20.32 nm). SEM and EDAX analyses confirmed uniform dispersion of NR and COE within the PHBV matrix. The analysis of water uptake kinetics revealed the suitability of both pseudo-first-order and pseudo-second-order models for describing water absorption behavior in the blends. Additionally, the equilibrium water uptake was ~62% higher for the PHBV/NR blend compared to those blended with COE, highlighting the significant impact of COE on reducing water absorption. Furthermore, the effects of COE on surface wettability were analyzed, resulting in an increased hydrophobicity. Future research should also explore the mechanical properties and scalability of these blends for their successful translation into practical applications. This work demonstrates the exciting potential of waste-derived COE in creating sustainable PHBV/NR blends with tailored properties for various uses.

## Figures and Tables

**Table 1:** Extrusion Temperature Profile.

Zone	T_1_	T_2_	T_3_	T_4_	T_5_	T_6_	T_7_	T_8_	T_9_	Die
Temperature (°C)	180	180	178	178	175	175	167	165	165	162

**Table 2:** Thermal properties of PHBV and its NR blend.

Sample	Δ*H_m_ (J/g)*	*T_m_ onset (°C) *	*T_m_ peak (°C) *	Δ*H_c_ (J/g) *	*T_c_ onset (°C) *	*T_c_ peak (°C) *	*X_c_*	*T_g_ of PHBV *	*T_g_ of Rubber *
PHBV	92.5 ± 0.3	163.6 ± 0.9	170.4 ± 0.9	93.6 ± 0.4	124.5 ± 0.3	120.9 ± 0.4	68.5 ± 0.5	2.6 ± 0.4	−
PHBV/NR	79.6 ± 1.2	160.8 ± 0.4	165.4 ± 0.2	78.3 ± 1.4	120.1 ± 0.2	116.4 ± 0.2	54.5 ± 0.8	1.8 ± 0.3	(−) 62.6 ± 1.3
PHBV/NR/COE	78.9 ± 1.0	160.7 ± 0.1	166.4 ± 0.2	77.3 ± 1.2	119.7 ± 0.1	115.9 ± 0.2	53.5 ± 0.4	1.7 ± 0.3	(−) 63.6 ± 1.4

Negative Tg values indicated by a (−) sign represent the glass transition temperature below 0 °C for Natural Rubber.

**Table 3:** Thermal degradation temperatures of PHBV and the blends.

	Thermal Degradation Temperature		Natural Rubber	
Samples	T Onset (°C)	T Peak (°C)	T Onset (°C)	T Peak (°C)
PHBV	294.6 ± 3.1	304.0 ± 4.5	-	-
PHBV/NR	298.8 ± 0.3	310.7 ± 0.9	328.0 ± 0.9	367.9 ± 0.6
PHBV/NR/COE	297.9 ± 2.4	308.6 ± 2.5	327.0 ± 3.0	369.0 ± 1.1

**Table 4:** Crystallographic parameters of PHBV/NR Blend with and without COE.

Sample	2θ (°)	hkl	d-Spacing (Å)	X_c_ (%)	FWHM (°)	D-Value (nm)
PHBV/NR	14.74	020	5.86	63.52	0.35	22.67
	18.16	110	4.76		0.45	17.66
	27.92	121	3.11		1.69	4.82
PHBV/NR/COE	13.58	020	6.51	68.85	0.39	20.32
	17.08	110	5.18		0.63	12.72
	26.92	121	3.31		0.34	23.73

**Table 5:** Kinetics studies of water uptake in PHBV/NR and PHBV/NR/COE blends.

Samples	Pseudo-First-Order Model			Pseudo-Second-Order Model			Intraparticle Diffusion Model		
	k_1_	q_e_	R^2^	k_2_	q_e_	R^2^	K_in_	C	R^2^
PHBV/NR	0.073	1.385	0.994	0.084	1.526	0.998	0.147	0.163	0.862
PHBV/NR/COE	0.023	0.382	0.999	0.032	0.543	0.989	0.035	9.89 × 10^−26^	0.976

**Table 6:** WVTR and OTR values of plasticized and non-plasticized blends.

Samples	WVTR	OTR
	g/m^2^.24 h	cc/m^2^/day
PHBV/NR	4.05 ± 0.54	2025.29 ± 35.69
PHBV/NR/COE	1.55 ± 0.20	21.51 ± 1.68

**Table 7:** Elemental composition obtained by EDAX analysis for neat PHBV, PHBV/NR, and PHBV/NR/COE blends, respectively.

Samples	Element	Weight %	Atomic %
PHBV	C	82.26	86.26
	O	17.18	13.52
	Ca	0.29	0.09
	Al	0.28	0.13
PHBV/NR	C	60.55	54.61
	O	3.67	2.49
	Ca	0.05	0.01
	Al	0.07	0.03
	Be	35.65	42.85
PHBV/NR/COE	C	69.64	75.71
	O	29.17	23.81
	Ca	0.59	0.19
	Al	0.59	0.29

## Data Availability

The data supporting the findings of this study are presented within the article and available upon request from the corresponding author.
